# Distal Versus Proximal Intracoronary Drug Delivery for the Treatment of the No-Reflow Phenomenon During Percutaneous Coronary Intervention

**DOI:** 10.3390/jcm15166252

**Published:** 2026-08-13

**Authors:** Umeyir Savur, Berhan Keskin, Irem Koc, Yerkenur Khidolda, Atakan Dursun, Aysel Akhundova, Aykun Hakgor, Haci Murat Gunes, Bilal Boztosun

**Affiliations:** Department of Cardiology, Istanbul Medipol University, Medipol Mega University Hospital, 34214 Istanbul, Türkiye

**Keywords:** no-reflow phenomenon, percutaneous coronary intervention, intracoronary drug delivery, thrombolysis in myocardial infarction (TIMI) flow, coronary microvascular obstruction

## Abstract

**Background/Objectives**: The optimal intracoronary drug delivery strategy for the management of the no-reflow phenomenon (NRP) during percutaneous coronary intervention (PCI) remains uncertain. We compared distal and proximal intracoronary drug administration for the treatment of NRP. **Methods**: This retrospective single-center study included 212 consecutive patients who developed NRP following drug-eluting stent implantation between January 2022 and June 2025. Patients were treated with intracoronary medications administered either proximally through the guiding catheter (*n* = 107) or distally using a microcatheter or guide extension catheter (*n* = 105). The primary outcome was the restoration of final Thrombolysis in Myocardial Infarction (TIMI) grade 3 flow. Multivariable logistic regression with bootstrap internal validation was performed. **Results**: Despite a higher prevalence of baseline TIMI 0 flow (85.7% vs. 19.6%, *p* < 0.001) and higher SYNTAX scores in the distal group, distal drug administration achieved a higher rate of final TIMI grade 3 flow (66.7% vs. 27.1%, *p* < 0.001) and final TIMI grade 2–3 flow (90.5% vs. 73.8%, *p* = 0.003). Distal administration remained an independent predictor of final TIMI grade 3 flow (adjusted OR 15.94, 95% CI 5.22–48.69; *p* < 0.001), with consistent findings in a sensitivity analysis accounting for adjunctive intracoronary pharmacological therapy. The optimism-corrected area under the receiver operating characteristic curve was 0.707. No significant between-group differences were observed in in-hospital or 6-month clinical outcomes. **Conclusions**: Distal intracoronary drug administration was associated with a higher likelihood of achieving TIMI grade 3 flow than conventional proximal administration. These findings should be considered hypothesis-generating and require confirmation in prospective randomized studies.

## 1. Introduction

The no-reflow phenomenon (NRP) remains a frequent complication of percutaneous coronary intervention (PCI) and is characterized by impaired myocardial perfusion despite successful restoration of epicardial coronary blood flow [1]. The pathophysiology of NRP is multifactorial and includes distal embolization, ischemia–reperfusion injury, endothelial dysfunction, microvascular vasoconstriction, interstitial edema, inflammatory injury, and the generation of reactive oxygen species [1,2,3]. Despite substantial advances in PCI techniques and adjunctive pharmacotherapy, NRP continues to be associated with adverse clinical outcomes, including increased mortality, adverse left ventricular remodeling, progression of heart failure, and ventricular arrhythmias [4,5,6].

Pharmacological therapy remains the cornerstone of NRP management in contemporary PCI practice. Several intracoronary agents, including adenosine, calcium channel blockers (verapamil and diltiazem), nitroglycerin, glycoprotein IIb/IIIa inhibitors, and epinephrine, are commonly used to treat NRP [1,3,7,8]. These agents primarily aim to alleviate microvascular vasoconstriction, improve microvascular perfusion, and reduce the impact of distal microvascular obstruction.

Despite the availability of multiple pharmacological agents, NRP may persist even after repeated administration of different drugs. In such cases, the method of intracoronary drug delivery may play an important role in treatment success. Although intracoronary drugs are commonly administered through the guiding catheter, this approach may not achieve adequate drug concentrations within the distal coronary microcirculation. Alternatively, distal intracoronary drug delivery can be performed using dedicated devices such as microcatheters, over-the-wire balloons, or guide extension catheters, allowing direct delivery of the drug to the affected microvascular territory [4,7]. Despite the widespread use of both proximal and distal drug delivery strategies in clinical practice, comparative data regarding their effectiveness remain limited.

Distal intracoronary drug delivery using dedicated devices has previously been described as a feasible strategy for the treatment of no-reflow during primary PCI, with encouraging angiographic results reported by Kumar et al. in a small cohort of patients with STEMI [9]. However, evidence remains limited, and the generalizability of these findings to broader PCI populations is uncertain. Moreover, comparative data evaluating distal versus conventional proximal intracoronary drug administration, together with their impact on echocardiographic and clinical outcomes, are lacking. Therefore, the present study aimed to evaluate the impact of the intracoronary drug delivery method (distal versus proximal administration) on angiographic treatment success in patients with NRP and to assess its echocardiographic and clinical implications in a contemporary real-world PCI cohort.

## 2. Materials and Methods

### 2.1. Study Population

**Figure 1:**
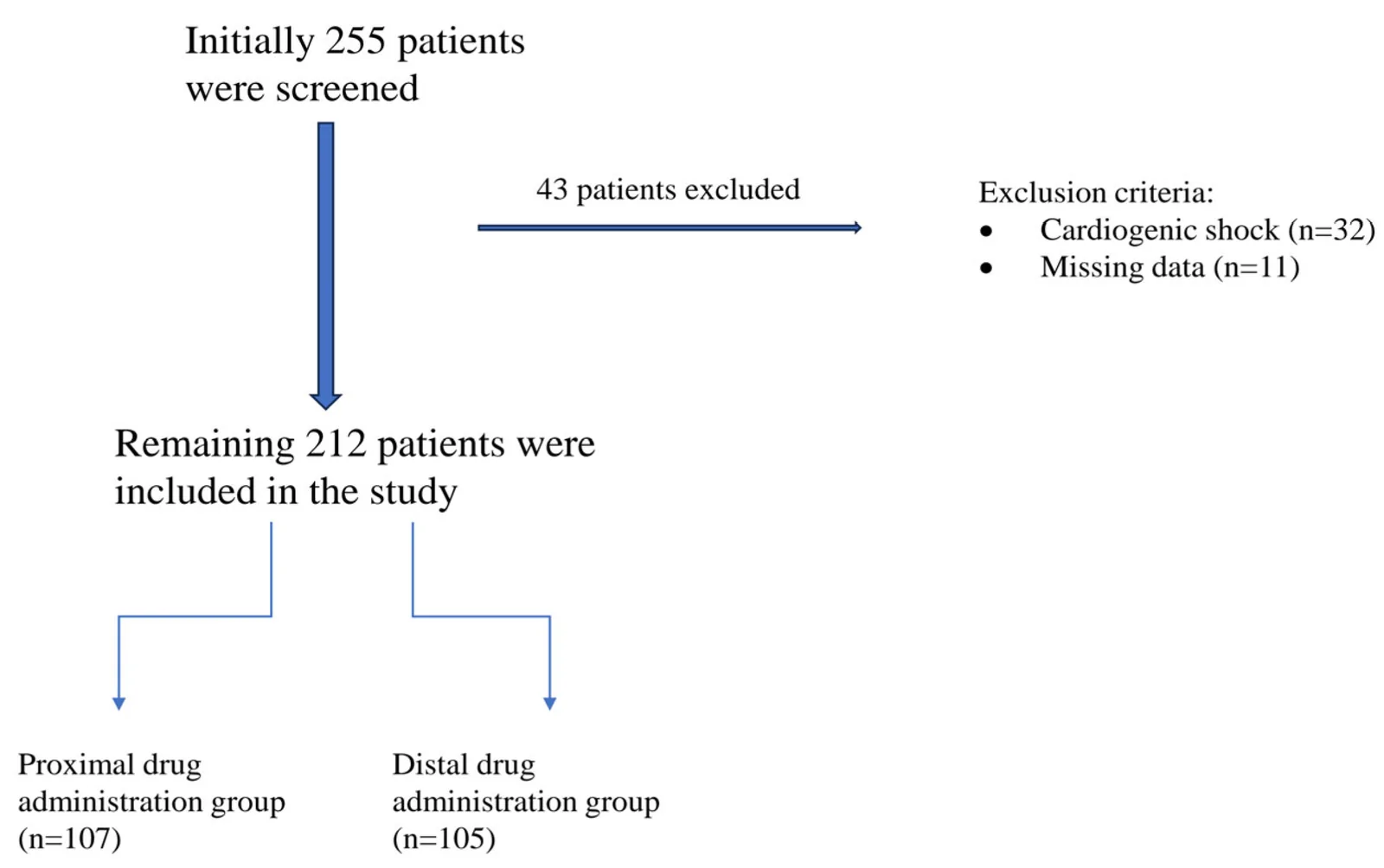
Patient flow diagram.

This single-center, retrospective study included consecutive adult patients who developed angiographically confirmed no-reflow or slow-flow phenomenon (Thrombolysis in Myocardial Infarction [TIMI] flow grade 0 or 1) following drug-eluting stent (DES) implantation and subsequently received intracoronary pharmacological treatment between 1 January 2022 and 1 June 2025. For the purposes of this study, baseline TIMI flow was defined as the TIMI flow grade immediately after the occurrence of no-reflow or slow-flow following stent implantation and immediately before intracoronary drug administration. Patients presenting with cardiogenic shock at admission or those with missing data were excluded (Figure 1). The remaining eligible patients constituted the study population.

### 2.2. Procedural Information and Treatment Protocol

All patients received a loading dose of aspirin together with a P2Y12 inhibitor (clopidogrel 600 mg, ticagrelor 180 mg, or prasugrel 60 mg) before the procedure. Lesion preparation was routinely performed using appropriately sized semi-compliant balloons. Non-compliant, cutting, or scoring balloons were used when necessary to optimize lesion preparation. Following DES implantation, post-dilation with non-compliant balloons was routinely performed to ensure optimal stent expansion. Intravascular imaging using optical coherence tomography (OCT) or intravascular ultrasound (IVUS) was not performed in any patient.

Patients with no-reflow or slow-flow phenomenon were treated with intracoronary medications administered either proximally through the guiding catheter or distally using a microcatheter or a guide extension catheter (GuideLiner^®^, Teleflex, Wayne, PA, USA). The choice of intracoronary drug delivery strategy (proximal versus distal) and the device used for distal administration was not protocol-driven and was left entirely to the discretion of the treating operator. No predefined angiographic or clinical criteria determined treatment allocation. In routine practice, the selected strategy reflected the operator’s real-time assessment of lesion characteristics, coronary flow, procedural complexity, and the perceived likelihood of achieving effective drug delivery to the distal coronary bed. According to the institutional treatment protocol, all patients initially received intracoronary nitroglycerin (100–300 μg, unless contraindicated because of hypotension) and adenosine (60–300 μg) following the occurrence of no-reflow or slow-flow. Patients with persistent no-reflow despite this initial therapy received intracoronary epinephrine (50–300 μg) as bailout treatment. Intracoronary tirofiban was administered at the operator’s discretion in patients with a high thrombus burden.

Following PCI, all patients received guideline-directed medical therapy, including dual antiplatelet therapy (DAPT) for at least 6 months, high-intensity statins, and other evidence-based therapies (beta-blockers, angiotensin-converting enzyme inhibitors or angiotensin receptor blockers, sodium-glucose cotransporter-2 inhibitors, and mineralocorticoid receptor antagonists) when clinically indicated.

### 2.3. Data Collection

Baseline demographic characteristics, comorbidities, laboratory findings, echocardiographic measurements, and angiographic data were retrospectively collected from the hospital information system and electronic medical records. Baseline clinical characteristics and laboratory parameters were recorded at hospital admission. Transthoracic echocardiography was performed by experienced cardiologists using a Vivid E95 ultrasound system (GE Vingmed Ultrasound, Milwaukee, WI, USA) equipped with a 3.5 MHz or M5S transducer. Left ventricular ejection fraction (LVEF) was estimated by visual assessment.

### 2.4. Outcomes

The primary outcome was the restoration of final TIMI grade 3 flow following intracoronary medication administration.

Secondary angiographic and in-hospital outcomes included the restoration of final TIMI grade 2–3 flow, a degree of TIMI flow improvement, definite in-hospital stent thrombosis according to the Academic Research Consortium (ARC) criteria [10], ventricular arrhythmias (non-sustained ventricular tachycardia, sustained ventricular tachycardia, or ventricular fibrillation occurring during the index hospitalization), acute kidney injury (AKI), and all-cause mortality. AKI was defined according to the Kidney Disease: Improving Global Outcomes (KDIGO) criteria as any of the following occurring after the index PCI procedure: an increase in serum creatinine of ≥0.3 mg/dL (≥26.5 μmol/L) within 48 h, an increase in serum creatinine to ≥1.5 times the baseline value within 7 days, or urine output < 0.5 mL/kg/h for at least 6 h [11].

Echocardiographic outcomes included LVEF, left ventricular end-diastolic diameter, left ventricular end-systolic diameter, left atrial diameter, tricuspid annular plane systolic excursion (TAPSE), and the presence of moderate or greater mitral, tricuspid, and aortic regurgitation at hospital discharge and at the 6-month follow-up.

Clinical outcomes during the 6-month follow-up included hospitalization for heart failure requiring intravenous diuretic therapy, repeat coronary revascularization, and all-cause mortality. Outcome data were obtained from the hospital information system and electronic medical records.

### 2.5. Statistical Analysis

Patients were categorized according to the intracoronary drug delivery strategy as proximal or distal administration. The distribution of continuous variables was assessed using visual inspection of histograms and the Kolmogorov–Smirnov test. Normally distributed continuous variables are presented as mean ± standard deviation (SD), whereas non-normally distributed variables are expressed as median and interquartile range (IQR; 25th–75th percentile). Categorical variables are presented as frequencies and percentages. Categorical variables were compared using the chi-square test or Fisher’s exact test, as appropriate. Continuous variables were compared using the independent-samples *t* test or the Mann–Whitney *U* test according to data distribution.

Predictors of the restoration of final TIMI grade 3 flow were evaluated using univariable and multivariable logistic regression analyses. Variables with a *p* value < 0.10 in the univariable analysis, together with clinically relevant covariates, were entered into the multivariable model. Odds ratios (ORs) with corresponding 95% confidence intervals (CIs) were reported.

Internal validation of the final multivariable model was performed using 1000 bootstrap resamples. Model discrimination was evaluated using both the apparent and optimism-corrected area under the receiver operating characteristic curve (AUC), whereas overall model performance was assessed using the Brier score.

All statistical tests were two-sided, and a *p* value < 0.05 was considered statistically significant. Statistical analyses were performed using Python version 3.14.0 (Python Software Foundation, Wilmington, DE, USA).

ChatGPT (OpenAI; GPT-5.6) was used solely for English-language editing to improve grammar, clarity, and readability of the manuscript. The tool was not used to generate study data, perform statistical analyses, or formulate scientific conclusions. All AI-assisted edits were critically reviewed and approved by the authors, who take full responsibility for the final content of the manuscript.

## 3. Results

A total of 212 patients who developed NRP following DES implantation were included in the study, comprising 107 patients in the proximal drug administration group and 105 patients in the distal drug administration group. Baseline demographic characteristics, clinical variables, and laboratory findings were generally comparable between the two groups (Table 1). Likewise, the use of in-hospital medications did not differ significantly between groups (Table 1).

**Figure 2:**
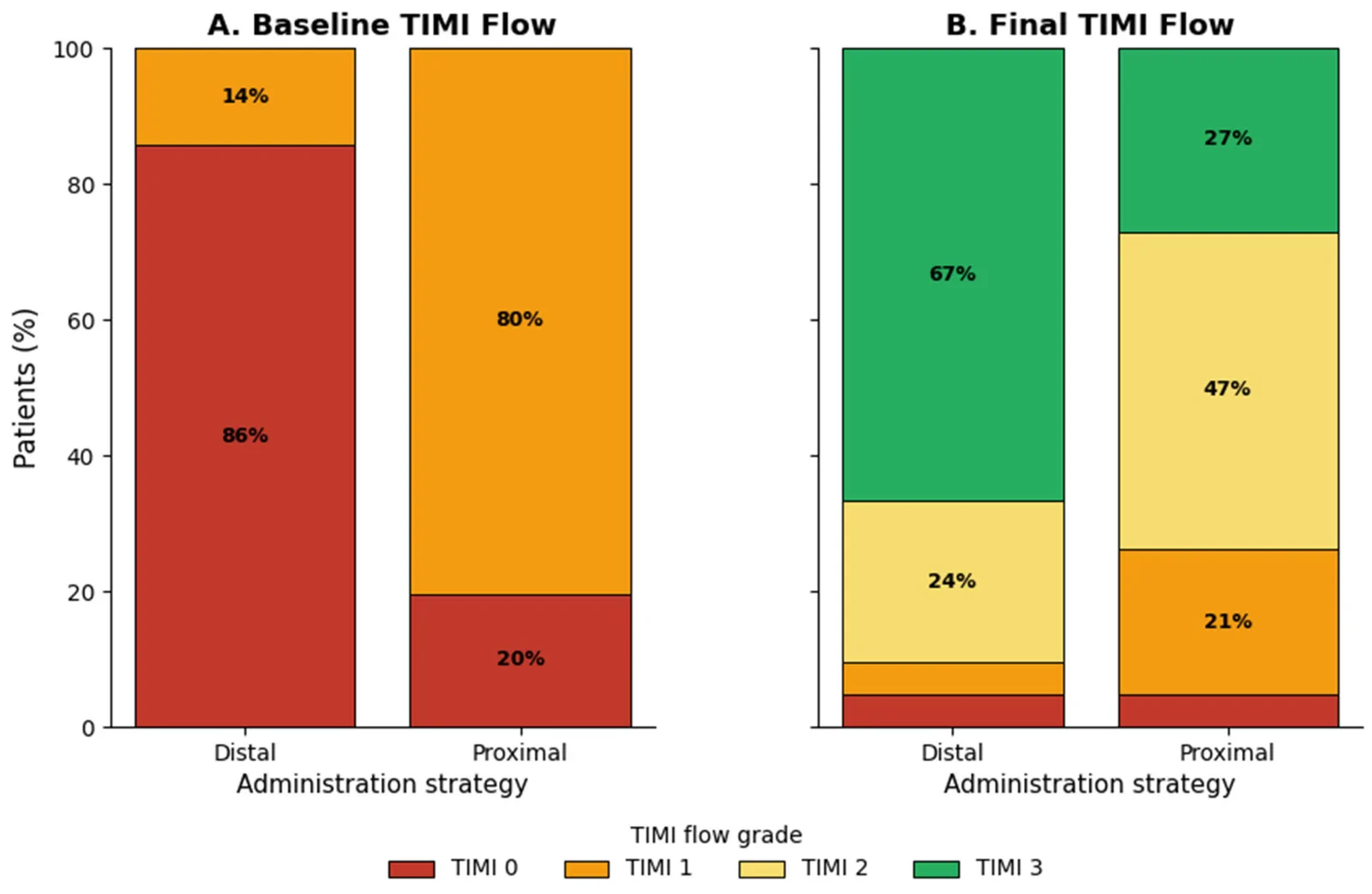
Baseline and final TIMI flow according to intracoronary drug administration strategy.

With respect to procedural and angiographic characteristics, the distribution of elective, non-ST-elevation myocardial infarction (NSTEMI), and ST-segment elevation myocardial infarction (STEMI) cases was similar between groups (Table 1). Patients in the distal drug administration group had more severe baseline coronary flow impairment, with a substantially higher prevalence of baseline TIMI 0 flow (85.7% vs. 19.6%, *p* < 0.001) (Table 1, Figure 2). The SYNTAX score was also higher in the distal administration group, indicating greater angiographic disease complexity (15.10 ± 6.45 vs. 12.85 ± 6.01, *p* = 0.036). Balloon and stent diameters, balloon and stent lengths, culprit lesion stenosis severity, and total occlusion rates were comparable between groups (Table 1).

Distal drug administration resulted in a significantly higher rate of final TIMI 3 flow than proximal administration (66.7% vs. 27.1%, *p* < 0.001) (Table 2, Figure 2). Likewise, the mean final TIMI flow grade (2.52 ± 0.80 vs. 1.96 ± 0.82, *p* < 0.001) and the proportion of patients achieving final TIMI 2–3 flow (90.5% vs. 73.8%, *p* = 0.003) were significantly greater in the distal administration group. Improvements in TIMI flow of at least one grade (95.2% vs. 78.5%, *p* < 0.001) and at least two grades (90.5% vs. 32.7%, *p* < 0.001) were also observed more frequently in patients receiving distal drug administration (Table 2). In contrast, other in-hospital outcomes, including stent thrombosis, post-procedural acute kidney injury, ventricular arrhythmias, and all-cause mortality, were similar between groups (Table 2).

Regarding echocardiographic outcomes, patients in the distal drug administration group had a lower discharge LVEF than those in the proximal administration group (45.45 ± 12.81% vs. 50.76 ± 11.13%, *p* = 0.016), whereas all other discharge echocardiographic parameters were comparable (Table 2). At the 6-month follow-up, LVEF did not differ significantly between groups (51.21 ± 10.49% vs. 52.08 ± 11.86%, *p* = 0.366). However, the improvement in LVEF from discharge to 6 months (ΔLVEF) was significantly greater in the distal administration group (3.80 ± 5.51% vs. 0.13 ± 5.05%, *p* < 0.001) (Table 2). No significant between-group differences were observed in the remaining 6-month echocardiographic parameters. Similarly, hospitalization for heart failure during follow-up occurred at comparable rates, and no patient underwent repeat coronary revascularization or died during the 6-month follow-up period (Table 2).

**Figure 3:**
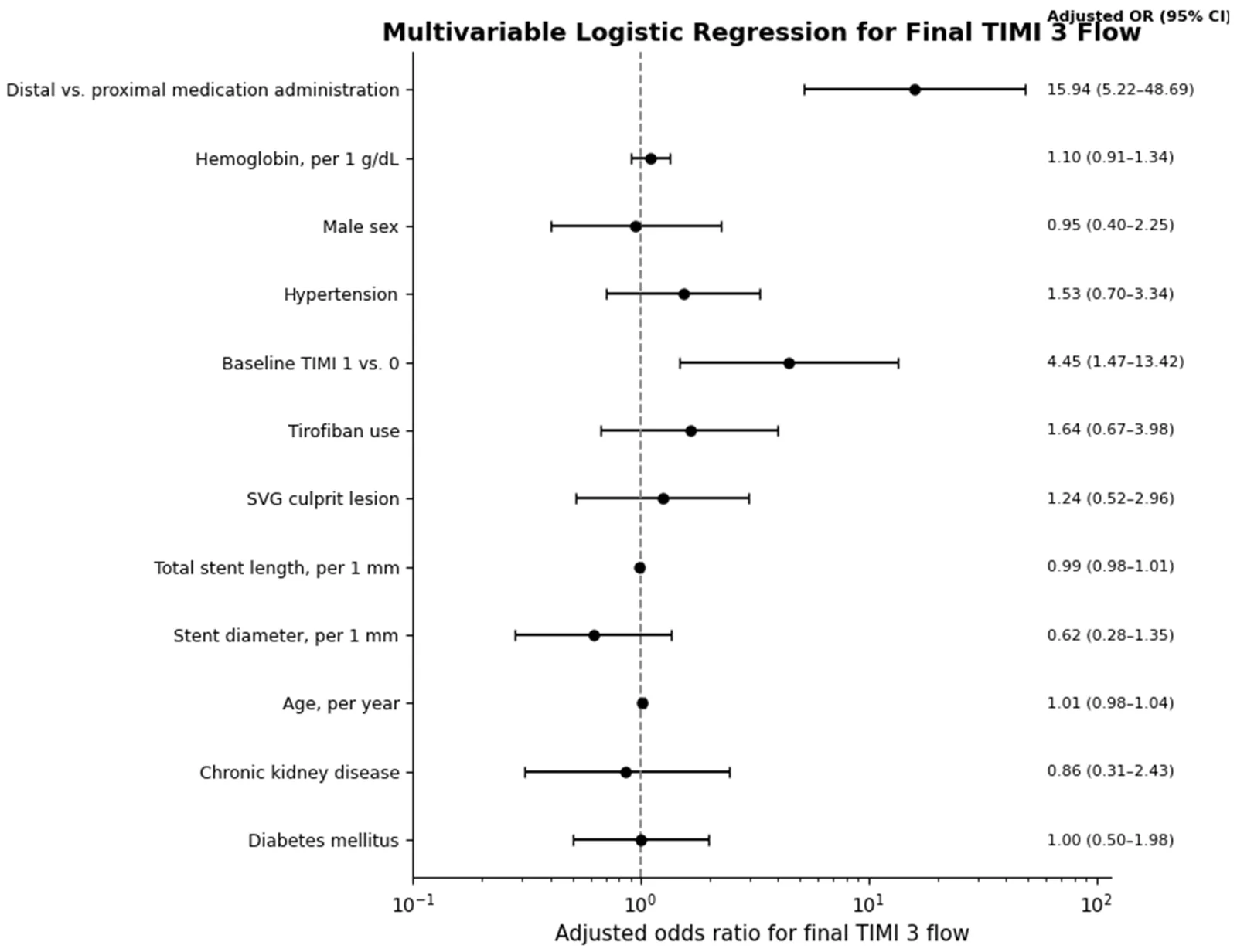
Forest plot of the multivariable logistic regression analysis for predictors of final TIMI grade 3 flow.

In the univariable logistic regression analysis evaluating predictors of final TIMI 3 flow, distal versus proximal drug administration (OR 5.379, 95% CI 2.986–9.690, *p* < 0.001), hypertension (OR 2.104, 95% CI 1.103–4.014, *p* = 0.024), and baseline TIMI 1 versus TIMI 0 flow (OR 0.535, 95% CI 0.309–0.924, *p* = 0.025) were significant predictors (Table 3). These variables, together with clinically relevant covariates, were subsequently entered into the multivariable logistic regression model. In the multivariable analysis, distal versus proximal drug administration (OR 15.942, 95% CI 5.219–48.693, *p* < 0.001) and baseline TIMI 1 versus TIMI 0 flow (OR 4.448, 95% CI 1.475–13.419, *p* = 0.008) remained independent predictors of successful restoration of final TIMI 3 flow (Table 4, Figure 3).

The multivariable model demonstrated good apparent discrimination, with an area under the receiver operating characteristic curve (AUC) of 0.759. Bootstrap internal validation yielded a mean optimism of 0.052, corresponding to an optimism-corrected AUC of 0.707, indicating acceptable discrimination after correction for overfitting. The apparent Brier score was 0.196, whereas the mean bootstrap Brier score was 0.209, supporting satisfactory overall model performance and internal stability.

In a sensitivity model including baseline TIMI flow and adjunctive intracoronary epinephrine and tirofiban use, distal drug administration remained strongly associated with the restoration of final TIMI grade 3 flow (adjusted OR 13.14, 95% CI 4.70–36.79; *p* < 0.001) (Supplementary Table S1).

Among patients who underwent distal drug administration, an exploratory subgroup analysis compared guide extension catheter (GuideLiner)-assisted delivery (*n* = 30) with microcatheter-assisted delivery (*n* = 75) (Supplementary Table S2). Patients treated using a guide extension catheter had a higher baseline TIMI flow grade than those treated with a microcatheter (TIMI 1 flow: 33.3% vs. 6.7%, *p* = 0.001). Guide extension catheter-assisted drug delivery was associated with a higher rate of final TIMI 3 flow (100.0% vs. 53.3%, *p* < 0.001) and a greater improvement in TIMI flow grade (2.67 ± 0.48 vs. 2.27 ± 0.86, *p* = 0.030). However, the rates of final TIMI 2–3 flow and TIMI flow improvement of at least two grades were similar between the two groups. Given the small sample size, the absence of outcome events in the guide extension catheter group, operator-dependent device selection, and baseline angiographic differences between groups, these findings should be interpreted with considerable caution and regarded as exploratory only.

## 4. Discussion

The principal finding of this study is that distal intracoronary drug administration using dedicated delivery devices, including microcatheters and guide extension catheters, was associated with superior restoration of coronary flow compared with conventional proximal drug administration through the guiding catheter in patients with the no-reflow phenomenon (NRP). Although numerous pharmacological agents have previously been investigated for the treatment of NRP, the impact of the drug delivery strategy itself has received little attention. Our findings suggest that, beyond drug selection, the method of intracoronary drug delivery represents an important determinant of treatment success in patients with NRP.

An interesting finding was the reversal in the direction of the association between baseline TIMI flow and the restoration of final TIMI 3 flow after multivariable adjustment. In the univariable analysis, baseline TIMI 1 vs. 0 flow appeared to be associated with a lower likelihood of achieving final TIMI 3 flow, whereas after adjustment it emerged as an independent positive predictor. This apparent paradox is most likely explained by confounding arising from the markedly unequal distribution of baseline TIMI flow between the treatment groups. Patients receiving distal drug administration had substantially worse initial coronary flow, with a much higher prevalence of baseline TIMI 0 flow, yet also experienced markedly higher rates of final TIMI 3 flow. Consequently, the strong treatment effect of distal drug delivery masked the favorable prognostic effect of baseline TIMI 1 flow in the unadjusted analysis. After accounting for treatment strategy and other covariates, the expected positive association between preserved baseline flow and successful reperfusion became evident. This finding highlights the importance of multivariable adjustment when comparing treatment strategies in observational studies with imbalanced baseline characteristics.

An additional consideration relates to the non-protocolized allocation of the intracoronary drug delivery strategy and the resulting baseline imbalances between the treatment groups. Because treatment selection was left entirely to the operator’s discretion, procedural judgment likely influenced the choice of proximal or distal drug administration. In patients with complete no-reflow (baseline TIMI 0), operators may have considered conventional proximal administration less likely to achieve adequate drug delivery to the distal microvascular bed because of the absence of antegrade flow, thereby favoring distal administration through a dedicated delivery device. Conversely, in patients with residual TIMI 1 flow, preserved antegrade perfusion may have been considered sufficient to allow effective distal drug distribution following proximal administration, reducing the perceived need for additional intracoronary devices. Consequently, patients in the distal administration group presented with a less favorable angiographic profile, including a substantially higher prevalence of baseline TIMI 0 flow and higher SYNTAX scores. Although these variables were accounted for in the adjusted analyses, the retrospective observational design precludes confirmation of the operators’ decision-making process, and residual confounding by indication cannot be excluded.

Intracoronary medications used for NRP exert their therapeutic effects through multiple mechanisms, including microvascular vasodilation, attenuation of the inflammatory response, and reduction in microvascular thrombotic burden [1,2,3]. Adenosine remains the most commonly used agent because of its vasodilatory, anti-inflammatory, and antiplatelet properties mediated through A2A receptor activation [1]. Tahirkeli et al. [12] suggested that prophylactic intracoronary adenosine administration before each balloon inflation or stent deployment may reduce the incidence of NRP. In contrast, the randomized trial by Baharvand et al. [13] demonstrated no reduction in NRP with prophylactic adenosine compared with standard treatment. Other agents, including nitroglycerin and calcium channel blockers, primarily improve microvascular perfusion through vasodilatory mechanisms. Intracoronary epinephrine has also been shown to be effective, particularly in refractory NRP and in patients with hypotension [2,3,8]. Although glycoprotein IIb/IIIa inhibitors have been associated with a lower incidence of NRP in previous studies [6], routine administration is not recommended because of the increased bleeding risk and the lack of consistent clinical benefit. Accordingly, current consensus documents recommend reserving these agents for patients with a substantial thrombus burden [1,2,3].

Despite the availability of several pharmacological therapies, approximately 20% of patients continue to exhibit persistent NRP despite treatment [1,2,3]. Although randomized trials evaluating sodium-glucose cotransporter-2 inhibitors for the prevention of NRP are currently ongoing [14], no pharmacological strategy has yet been proven to effectively prevent this complication. In our study, patients were managed according to a standardized institutional treatment algorithm consisting of intracoronary nitroglycerin (except in hypotensive patients) and adenosine as first-line therapy, followed by intracoronary epinephrine for refractory cases. Tirofiban was administered selectively in patients with a high thrombus burden. While previous investigations have primarily focused on comparing different pharmacological agents, our findings demonstrate that the delivery strategy itself is an additional and clinically relevant component of NRP management. Notably, although the pharmacological agents and their frequency of use were comparable between the two groups, distal drug administration resulted in substantially better angiographic outcomes than proximal administration.

These observations are consistent with the findings of the recent network meta-analysis by Oliveri et al. [15], which included 13 randomized trials comparing commonly used intracoronary vasoactive agents for the treatment of no-reflow during primary PCI. In that analysis, both verapamil and epinephrine were associated with significantly higher odds of achieving final TIMI grade 3 flow, whereas no pharmacological strategy demonstrated a significant reduction in major adverse cardiovascular events or mortality. Importantly, whereas the study by Oliveri et al. [15] focused on the comparative efficacy of different vasoactive agents, our study addresses a complementary and largely unexplored aspect of no-reflow management by evaluating the impact of the intracoronary drug delivery strategy itself. Together, these findings suggest that both the choice of pharmacological agent and the method of drug delivery may influence angiographic reperfusion success and warrant further prospective investigation.

Several predictors of NRP have previously been identified, including advanced age, diabetes mellitus, high thrombus burden, baseline TIMI 0 flow, heart failure, saphenous vein graft lesions, longer stent length, and increased inflammatory activity [1,6,16,17]. In the present study, the proximal and distal administration groups were generally well balanced with respect to these risk factors. Interestingly, patients undergoing distal drug administration had a higher prevalence of baseline TIMI 0 flow and higher SYNTAX scores, suggesting a greater angiographic risk profile. Nevertheless, these patients achieved significantly better final coronary flow than those treated with proximal administration. Furthermore, distal drug administration remained an independent predictor of the successful restoration of TIMI grade 3 flow after adjustment for clinically relevant variables, supporting the robustness of the observed association.

Another important finding was the greater improvement in left ventricular ejection fraction (LVEF) observed in the distal drug administration group during the 6-month follow-up. The detrimental impact of NRP on subsequent left ventricular systolic function has been consistently demonstrated in previous studies [6]. Therefore, the superior restoration of coronary microvascular perfusion achieved with distal drug administration may explain the greater recovery in LVEF observed in our cohort. Nevertheless, because of the retrospective design of the study, residual confounding cannot be excluded and these findings should be interpreted cautiously. Although NRP has previously been associated with adverse long-term outcomes, including heart failure hospitalization, repeat coronary revascularization, and mortality, our study did not demonstrate significant differences in these clinical endpoints. For example, a multicenter Australian registry including more than 18,000 PCI procedures reported significantly higher 3-year and 5-year all-cause mortality among patients who developed NRP [18]. The absence of differences in long-term clinical outcomes in our study is likely attributable to the relatively limited sample size and short follow-up duration. Moreover, our study was not specifically designed or powered to evaluate these secondary clinical outcomes.

Most previous studies evaluating distal intracoronary drug delivery have focused exclusively on patients with ST-segment elevation myocardial infarction. However, NRP also occurs in approximately 2.4% of elective PCI procedures [18]. Kumar et al. [9] demonstrated superior final TIMI flow with distal drug delivery using either a microcatheter or a perforated balloon compared with conventional proximal administration in patients with STEMI. Our study extends these findings by including patients with STEMI, NSTEMI, and elective PCI, thereby reflecting a more heterogeneous real-world population. Furthermore, unlike previous studies, we also evaluated short-term echocardiographic recovery and 6-month clinical outcomes, providing additional insight into the potential implications of successful NRP treatment beyond the procedural setting.

The limitations of the currently available evidence have also been highlighted by Beijnink et al. [19], who emphasized that the study by Kumar et al. [9] was retrospective, non-randomized, and primarily focused on angiographic reperfusion rather than myocardial infarct size or other markers of myocardial injury. These limitations should also be considered when interpreting our findings. Although the present study includes a larger and more heterogeneous real-world PCI population, compares distal and proximal drug delivery strategies, and additionally evaluates echocardiographic and clinical outcomes, its retrospective observational design precludes definitive conclusions regarding causality. Prospective randomized studies incorporating imaging- or biomarker-based measures of myocardial injury will be important to determine whether improved angiographic reperfusion translates into meaningful myocardial salvage and better clinical outcomes.

Another novel aspect of the present study is the exploratory evaluation of guide extension catheters as a platform for distal intracoronary drug delivery. Previous studies have predominantly investigated microcatheters and perforated balloon techniques, whereas evidence regarding guide extension catheters remains scarce [7]. Although all 30 patients treated with guide extension catheter-assisted drug delivery achieved final TIMI grade 3 flow, this observation should be interpreted with substantial caution because of the small sample size, the absence of outcome events in this subgroup, and the operator-dependent, non-randomized selection of the delivery device. The larger inner lumen of guide extension catheters, compared with microcatheters or perforated balloons, may permit delivery of a greater drug volume with higher injection pressure, potentially facilitating more effective distribution within the distal coronary microcirculation. Nevertheless, guide extension catheter use has been associated with potential complications, including coronary dissection, vessel perforation, and stent deformation or dislodgement [20]. Importantly, no device-related procedural or vascular complications were observed in our study. However, the present study was not designed or adequately powered to compare different distal drug delivery devices, and baseline angiographic differences further limit any comparative interpretation. Therefore, these findings should be regarded as hypothesis-generating only and should not be interpreted as evidence of superiority of guide extension catheters over microcatheters. Larger prospective comparative studies are required to validate these preliminary observations and to define the optimal delivery strategy for intracoronary pharmacotherapy in patients with NRP.

## 5. Conclusions

In this retrospective observational study, distal intracoronary drug administration was associated with a higher likelihood of achieving final TIMI grade 3 flow than conventional proximal administration in patients with the no-reflow phenomenon following PCI. Although these findings suggest that the method of intracoronary drug delivery may influence angiographic reperfusion, they should be interpreted cautiously given the observational study design. Prospective randomized studies are required to confirm these findings and determine whether improved angiographic reperfusion translates into better clinical outcomes.

## 6. Limitations

This study has several limitations. First, its retrospective, single-center design introduces the potential for selection bias and residual confounding despite multivariable adjustment and bootstrap internal validation. Furthermore, because the choice of proximal or distal drug administration, as well as the selection of the distal delivery device, was left to the operator’s discretion rather than a predefined protocol, confounding by indication cannot be excluded. Procedural strategies, including lesion preparation and the use of pre- and post-dilation, were likewise not standardized and may have influenced the occurrence and severity of the no-reflow phenomenon. Second, intravascular imaging with optical coherence tomography or intravascular ultrasound was not routinely performed; therefore, procedural factors such as stent under-expansion or edge dissection could not be systematically evaluated. Third, the sample size and the relatively short follow-up period limited the statistical power to detect differences in infrequent clinical outcomes, including mortality and repeat coronary revascularization. Finally, the exploratory analysis comparing guide extension catheter- and microcatheter-assisted drug delivery should be interpreted cautiously because of the limited sample size and baseline differences between groups. Prospective randomized multicenter studies are warranted to validate our findings.

## Figures and Tables

**Table 1:** Comparison of Proximal and Distal Intracoronary Medication Administration Groups in Patients with the No-Reflow Phenomenon.

Variable	Proximal (*n* = 107)	Distal (*n* = 105)	*p* Value
**Demographic and Clinical Characteristics**			
Age, years	62.29 ± 11.18	64.05 ± 12.34	0.137
Male sex	87 (81.3%)	80 (76.2%)	0.457
Hypertension	68 (63.6%)	78 (74.3%)	0.091
Chronic kidney disease	12 (11.2%)	19 (18.1%)	0.177
Diabetes mellitus	38 (35.5%)	45 (42.9%)	0.340
Atrial fibrillation	18 (16.8%)	13 (12.4%)	0.438
Previous PCI	23 (21.5%)	38 (36.2%)	0.023
Previous CABG	17 (15.9%)	25 (23.8%)	0.202
COPD	2 (1.9%)	6 (5.7%)	0.168
Congestive heart failure	30 (28.0%)	30 (28.6%)	1.000
Prior stroke	2 (1.9%)	7 (6.7%)	0.100
Peripheral artery disease	4 (3.7%)	5 (4.8%)	0.747
**Laboratory Parameters**			
Hemoglobin, mg/dL	14.21 ± 1.83	13.72 ± 2.12	0.082
White blood cell count, ×10^3^/µL	9.96 ± 3.82	9.46 ± 2.63	0.491
Platelet count, ×10^3^/µL	240.92 ± 66.47	237.10 ± 56.74	0.807
Creatinine, mg/dL	0.92 (0.80–1.10)	0.86 (0.82–1.05)	0.298
Urea, mg/dL	32.80 (27.15–43.12)	36.00 (28.00–49.00)	0.221
AST, U/L	21.50 (17.00–39.73)	30.00 (20.00–40.00)	0.382
ALT, U/L	20.50 (14.38–35.00)	43.00 (10.00–76.00)	0.894
LDL cholesterol, mg/dL	114.45 ± 45.89	110.56 ± 57.41	0.103
C-reactive protein, mg/L	5.93 (2.14–16.91)	4.01 (1.07–4.64)	0.157
**Intracoronary Medications for NRP**			
Adenosine	107 (100.0%)	105 (100.0%)	1.000
Nitroglycerine	99 (92.5%)	96 (91.4%)	0.801
Epinephrine	46 (43.0%)	37 (35.2%)	0.263
Tirofiban	23 (21.5%)	20 (19.0%)	0.785
**Medical Treatment (In-hospital)**			
ACE inhibitor/ARB	58 (54.2%)	66 (62.9%)	0.221
Statin	101 (94.4%)	100 (95.2%)	1.000
Beta-blocker	81 (75.7%)	88 (83.8%)	0.172
Insulin	11 (10.3%)	10 (9.5%)	1.000
SGLT2 inhibitor	18 (16.8%)	30 (28.6%)	0.060
Calcium-channel blocker	26 (24.3%)	37 (35.2%)	0.081
P2Y12 inhibitor			
Ticagrelor	22 (20.6%)	15 (14.3%)	0.221
Prasugrel	21 (19.6%)	30 (28.6%)	
Clopidogrel	64 (59.8%)	60 (57.1%)	
**Procedural and Angiographic Characteristics**			
Elective/NSTEMI/STEMI			
Elective	45 (42.1%)	45 (42.9%)	0.737
NSTEMI	35 (32.7%)	38 (36.1%)	
STEMI	27 (25.2%)	22 (21.0%)	
Baseline TIMI flow			
TIMI 0	21 (19.6%)	90 (85.7%)	<0.001
TIMI 1	86 (80.4%)	15 (14.3%)	
Culprit vessel			
LAD	33 (30.8%)	38 (36.2%)	0.446
CX	15 (14.0%)	15 (14.3%)	
DX/OM	2 (1.9%)	1 (1.0%)	
RCA	45 (42.1%)	33 (31.4%)	
SVG	12 (11.2%)	18 (17.1%)	
Balloon diameter, mm	2.66 ± 0.50	2.65 ± 0.44	0.916
Balloon length, mm	16.91 ± 3.00	17.10 ± 3.05	0.522
Stent diameter, mm	3.10 ± 0.42;	3.18 ± 0.47	0.348
Total culprit-vessel stent length, mm	38.00 (33.00–53.50)	48.00 (38.00–66.00)	0.051
Culprit lesion stenosis, %	92.28 ± 8.15	94.00 ± 5.40	0.483
SYNTAX score	12.85 ± 6.01	15.10 ± 6.45	0.036
Total occlusion	34 (31.8%)	35 (33.3%)	0.924

Abbreviations: ACE, angiotensin-converting enzyme; ALT, alanine aminotransferase; ARB, angiotensin II receptor blocker; AST, aspartate aminotransferase; CABG, coronary artery bypass grafting; COPD, chronic obstructive pulmonary disease; CX, left circumflex artery; DX, diagonal artery; LAD, left anterior descending artery; LDL, low-density lipoprotein; NRP, no-reflow phenomenon; NSTEMI, non-ST-segment elevation myocardial infarction; OM, obtuse marginal artery; PCI, percutaneous coronary intervention; RCA, right coronary artery; SGLT2, sodium-glucose cotransporter-2; STEMI, ST-segment elevation myocardial infarction; SVG, saphenous vein graft; SYNTAX, Synergy Between Percutaneous Coronary Intervention with Taxus and Cardiac Surgery; TIMI, Thrombolysis in Myocardial Infarction.

**Table 2:** Comparison of Outcomes According to Proximal and Distal Intracoronary Medication Administration in Patients with the No-Reflow Phenomenon.

Variable	Proximal (*n* = 107)	Distal (*n* = 105)	*p* Value
**In-Hospital Outcomes**			
Stent thrombosis	0 (0.0%)	0 (0.0%)	1.000
Post-procedural AKI	4 (3.7%)	8 (7.6%)	0.244
Ventricular arrhythmia	2 (1.9%)	4 (3.8%)	0.441
In-hospital mortality	1 (0.9%)	0 (0.0%)	1.000
Final TIMI flow grade	1.96 ± 0.82	2.52 ± 0.80	<0.001
Final TIMI 3 flow	29 (27.1%)	70 (66.7%)	<0.001
Final TIMI 2–3 flow	79 (73.8%)	95 (90.5%)	0.003
TIMI flow improvement ≥ 1 grade	84 (78.5%)	100 (95.2%)	<0.001
TIMI flow improvement ≥ 2 grades	35 (32.7%)	95 (90.5%)	<0.001
**Discharge Echocardiographic Outcomes**			
Discharge LVEF, %	50.76 ± 11.13	45.45 ± 12.81	0.016
Discharge LVEDD, cm	4.98 ± 0.48	5.21 ± 0.58	0.183
Discharge LVESD, cm	3.52 ± 0.63	3.58 ± 0.65	0.503
Discharge LA diameter, cm	3.99 ± 0.54	4.11 ± 0.40	0.127
Discharge TAPSE, cm	1.70 ± 0.42	1.80 ± 0.21	0.763
Discharge > moderate MR	13 (12.1%)	14 (13.3%)	0.810
Discharge > moderate TR	2 (2.7%)	0 (0.0%)	0.193
Discharge > moderate AR	0 (0.0%)	0 (0.0%)	1.000
**Six-Month Echocardiographic Outcomes**			
6-month LVEF, %	52.08 ± 11.86	51.21 ± 10.49	0.366
ΔLVEF from discharge to 6 months	0.13 ± 5.05	3.80 ± 5.51	<0.001
6-month LVEDD, cm	4.98 ± 0.52	5.14 ± 0.44	0.089
6-month LVESD, cm	3.52 ± 0.67	3.52 ± 0.40	0.576
6-month LA diameter, cm	3.96 ± 0.53	3.91 ± 0.30	0.698
6-month TAPSE, cm	2.00 ± 0.36	2.13 ± 0.27	0.836
6-month > moderate MR	12 (11.2%)	12 (11.4%)	0.964
6-month > moderate TR	2 (2.7%)	0 (0.0%)	0.193
6-month > moderate AR	0 (0.0%)	0 (0.0%)	1.000
**Six-Month Clinical Outcomes**			
6-month HF hospitalization	2 (1.9%)	2 (1.9%)	1.000
Coronary re-intervention	0 (0.0%)	0 (0.0%)	1.000
Mortality	0 (0.0%)	0 (0.0%)	1.000

Abbreviations: AKI, acute kidney injury; AR, aortic regurgitation; HF, heart failure; LA, left atrial; LVEDD, left ventricular end-diastolic diameter; LVEF, left ventricular ejection fraction; LVESD, left ventricular end-systolic diameter; MR, mitral regurgitation; TAPSE, tricuspid annular plane systolic excursion; TIMI, Thrombolysis in Myocardial Infarction; TR, tricuspid regurgitation; Δ, change.

**Table 3:** Univariable Logistic Regression Analysis for Predictors of Final TIMI 3 Flow.

Variables	Odds Ratio (OR)	95% CI	*p* Value
Distal vs. proximal medication administration	5.379	2.986–9.690	<0.001
Hypertension	2.104	1.103–4.014	0.024
Baseline TIMI 1 vs. TIMI 0	0.535	0.309–0.924	0.025
Tirofiban use	1.583	0.807–3.106	0.182
White blood cell count	0.946	0.868–1.031	0.207
C-reactive protein	0.981	0.948–1.014	0.249
Balloon length, per 1 mm	0.951	0.868–1.042	0.279
Balloon diameter, per 1 mm	0.747	0.414–1.345	0.330
Diabetes mellitus	1.294	0.744–2.250	0.361
LDL cholesterol, per 1 mg/dL	1.003	0.997–1.008	0.365
Insulin use	0.676	0.268–1.706	0.407
Previous CABG	1.329	0.675–2.614	0.411
Chronic kidney disease	1.354	0.637–2.878	0.430
SVG culprit lesion	1.354	0.637–2.878	0.430
Age, per year	1.008	0.985–1.032	0.482
Male sex	0.799	0.413–1.544	0.504
Total lesion vs. subtotal/none	0.825	0.463–1.471	0.514
ACE inhibitor/ARB use	1.193	0.686–2.074	0.531
AST, per 1 U/L	1.004	0.992–1.016	0.551
Peripheral artery disease	1.449	0.378–5.555	0.588
Stent diameter, per 1 mm	0.886	0.484–1.621	0.695
Atrial fibrillation	1.165	0.526–2.580	0.707
Hemoglobin, per 1 g/dL	0.977	0.851–1.122	0.743
Total stent length, per 1 mm	0.999	0.989–1.010	0.918
Culprit lesion stenosis, per 1%	1.002	0.964–1.042	0.922
SYNTAX score, per 1 point	1.000	0.952–1.051	0.998

Abbreviations: ACE, angiotensin-converting enzyme; ARB, angiotensin II receptor blocker; AST, aspartate aminotransferase; CABG, coronary artery bypass grafting; CI, confidence interval; LDL, low-density lipoprotein; OR, odds ratio; SVG, saphenous vein graft; SYNTAX, Synergy Between Percutaneous Coronary Intervention with Taxus and Cardiac Surgery; TIMI, Thrombolysis in Myocardial Infarction.

**Table 4:** Multivariable Logistic Regression Analysis for Predictors of Final TIMI 3 Flow.

Variable	Adjusted OR	95% CI	*p* Value
Distal vs. proximal medication administration	15.942	5.219–48.693	<0.001
Hemoglobin, per 1 g/dL	1.103	0.910–1.337	0.316
Male sex	0.952	0.403–2.246	0.910
Hypertension	1.526	0.697–3.345	0.291
Baseline TIMI 1 vs. 0	4.448	1.475–13.419	0.008
Tirofiban use	1.635	0.671–3.983	0.279
SVG culprit lesion	1.244	0.522–2.965	0.623
Total stent length, per 1 mm	0.994	0.981–1.007	0.353
Stent diameter, per 1 mm	0.619	0.284–1.346	0.226
Age, per year	1.011	0.981–1.042	0.477
Chronic kidney disease	0.864	0.306–2.434	0.781
Diabetes mellitus	0.999	0.504–1.981	0.998

Abbreviations: CI, confidence interval; OR, odds ratio; SVG, saphenous vein graft; TIMI, Thrombolysis in Myocardial Infarction.

## Data Availability

The datasets generated during and/or analyzed during the current study are not publicly available, but they are available from the corresponding author on reasonable request.

## Supplementary Materials

The following supporting information can be downloaded at https://www.mdpi.com/article/10.3390/jcm15166252/s1, Table S1. Multivariable logistic regression model including intracoronary pharmacological therapies for the prediction of final TIMI grade 3 flow; Table S2. Angiographic outcomes according to the distal drug delivery device among patients undergoing distal intracoronary medication administration.

## Abbreviations

The following abbreviations are used in this manuscript:

NRP	No-reflow phenomenon
PCI	Percutaneous coronary intervention
TIMI	Thrombolysis in myocardial infarction
OCT	Optical coherence tomography
IVUS	Intravascular ultrasound
DAPT	Dual antiplatelet therapy
LVEF	Left ventricular ejection fraction
AKI	Acute kidney injury
SD	Standard deviation
IQR	Interquartile range
OR	Odds ratio
CI	Confidence interval
AUC	Area under curve
NSTEMI	Non-ST-segment elevation myocardial infarction
STEMI	ST-segment elevation myocardial infarction
