# Supplementary material for: Distal Versus Proximal Intracoronary Drug Delivery for the Treatment of the No-Reflow Phenomenon During Percutaneous Coronary Intervention
Source: J Clin Med. 2026 Aug 13;15(16):6252. doi: 10.3390/jcm15166252 (PMC13513487; doi:10.3390/jcm15166252)
Supplement: Supplementary file 1 [file jcm-15-06252-s001.zip › jcm-4466571-supplementary.pdf]

Supplementary Tables

Supplementary Table S1. Multivariable logistic regression model including intracoronary pharmacological therapies for the prediction of final TIMI grade 3 flow.

Variable	Adjusted OR	95% CI	P value
Distal vs. proximal drug administration	**13.14**	**4.70-36.79**	**<0.001**
Baseline TIMI 1 vs. TIMI 0 flow	**3.57**	**1.27-10.01**	**0.016**
Epinephrine use	1.52	0.80-2.91	0.202
Tirofiban use	2.31	1.03-5.17	0.042

Abbreviations: CI, confidence interval; CKD, chronic kidney disease; OR, odds ratio; SVG, saphenous vein graft; TIMI, Thrombolysis in Myocardial Infarction.

Supplementary Table S2. Angiographic outcomes according to the distal drug delivery device among patients undergoing distal intracoronary medication administration.

Variable	Guide extension catheter (n=30)	Microcatheter (n=75)	P value
**Baseline TIMI flow**			**0.001**
TIMI 0	20 (66.7)	70 (93.3)	
TIMI 1	10 (33.3)	5 (6.7)	
**Final TIMI flow**			**<0.001**
TIMI 0	0 (0.0)	5 (6.7)	
TIMI 1	0 (0.0)	5 (6.7)	
TIMI 2	0 (0.0)	25 (33.3)	
TIMI 3	30 (100.0)	40 (53.3)	
Final TIMI flow, mean ± SD	3.00 ± 0.00	2.33 ± 0.88	<0.001
TIMI flow improvement, mean ± SD	2.67 ± 0.48	2.27 ± 0.86	0.030
Final TIMI 3 flow	30 (100.0)	40 (53.3)	<0.001
Final TIMI 2-3 flow	30 (100.0)	65 (86.7)	0.059
TIMI flow improvement ≥1 grade	30 (100.0)	70 (93.3)	0.318
TIMI flow improvement ≥2 grades	30 (100.0)	65 (86.7)	0.059

Abbreviations: TIMI, Thrombolysis in Myocardial Infarction.
